# Corporate income tax, IP boxes and the location of R&D

**DOI:** 10.1007/s10797-023-09812-x

**Published:** 2024-01-10

**Authors:** Pranvera Shehaj, Alfons J. Weichenrieder

**Affiliations:** 1 Freie Universität Berlin, School of Business and Economics, 14195 Berlin, Germany; 2 Faculty of Economics and Business, Goethe University Frankfurt, 60323 Frankfurt (Main), Germany; 3 Vienna University of Economics and Business, Vienna, Austria; 4 SAFE, Frankfurt (Main), Germany; 5 CESifo, Munich, Germany

**Keywords:** Corporate income tax, R&D, Intellectual property regimes, Patent box, International profit shifting, H25, H26, O3

## Abstract

We discuss corporate tax effects on multinationals’ R&D. Theoretically, we find that a host country’s tax increase may boost local R&D expenditure: while R&D becomes deductible at a higher rate, this higher rate may not apply to all R&D returns. First, as R&D creates a public good within the MNE, some R&D returns are taxed at other countries’ tax rates. Second, some of the R&D returns are taxed at a lower IP regime tax rate. The positive tax rate effect is empirically supported by country-by-country R&D data of U.S.-owned subsidiaries for countries that have an IP regime.

## Introduction

The taxation of income from intellectual property (IP) has received considerable research interest in recent years. Much of this interest has been stirred by new tax legislation and the political debate connected to it. In the last twenty years, many countries have moved to schedular taxation by introducing a special tax rate on intellectual property income that is below the standard corporate tax rate. Schemes that introduce such a schedularization of business taxation have been labeled as patent boxes, innovation boxes, IP boxes, or IP regimes (OECD, 2015, 2017).

Intangible assets are increasingly perceived to be important value-drivers within multinational enterprises (MNEs). From the perspective of an MNE, locating these assets in low-tax affiliates is an attractive tax-saving strategy. There is a large body of literature, more extensively reviewed in the next section, that indicates that IP boxes and taxes on income from intellectual property affect the location of patents. For example, Griffith et al. (2014) and Alstadsæter et al. (2018) provide evidence that a lower tax on IP income increases the number of patents that are registered in the respective jurisdiction.

While there is ample evidence that low tax rates attract the location of patents, empirical evidence on the effects of taxes on the location of actual research and development (R&D) activity has received less attention. An exception is Alstadsæter et al. (2018) who find that the tax advantage of IP boxes in a panel sample of large research-intensive multinationals is negatively rather than positively correlated with a variable designed to measure the shift of inventors to IP box countries. A further notable exception is Hines (1993, 1994) who uses data on U.S. multinationals to analyze the changed tax incentives from the U.S. 1986 tax reform and further reforms between 1986 and 1990. While Hines considers changes of R&D in the U.S., the present paper is concerned with R&D performed in foreign affiliates of U.S. multinationals.

One difficulty of identifying tax effects on the location of R&D activity is that country-level data on R&D expenditures of multinational companies is scarce. Most existing studies on IP boxes work with patent registration data. At the same time, the location of patent registration provides limited evidence on where the actual R&D activities take place and, in particular, on the size of local R&D expenditures. When it comes to patent registration data, evidence on the distinction of patent registration and the location of real R&D activity can only be derived from a gap between the country of registration and the residence country of the inventor. This leaves a potentially important research gap since the technological spillover effects of foreign direct investment and foreign know-how are expected to result from the size of real research activity, not so much from the mere registration of patents or the residence of single individuals. Within an MNE, the registration of patents may be influenced by the wish to shift profit into low-tax jurisdictions, without much connection to the location of research activity.

While corporate groups report aggregate R&D expenditures, the distribution across different subsidiaries is difficult to obtain in data bases that are readily available for researchers. The present paper adds empirical evidence on real R&D activity by looking at R&D expenditures of U.S. majority-owned subsidiaries abroad, data that are available on the country-year level from the Bureau of Economic Analysis (BEA).

Our empirical analysis is guided by a model of optimal R&D decisions. Within our model MNE, the benefits of R&D derive from royalty income and from increased productivity of the MNE as a whole. Since the increased productivity is not confined to the subsidiary that undertakes the R&D, research expenditures contribute toward a public good within the MNE.^1^ This can have interesting and counter-intuitive implications for the role of corporate taxes if one country exogenously increases its corporate tax. An increase in the corporate tax of one country means that the value of the cost deductibility of R&D expenditures increases in this country. This effect is similar to the well-known debt-tax shield, which incentivizes corporations to use more tax-deductible interest if the corporate tax is higher: with a higher tax rate the value of interest deductions is more valuable, while the tax of those who receive the interest may be unchanged. Increasing R&D expenditures when the tax is higher may also lead to a higher tax on the benefits of R&D. This effect, however, is limited within an MNE. Due to the public goods characteristics of R&D, the additional benefits of a marginal unit of R&D occurs in other countries as well. As the tax rates of these other countries are constant, the average tax rate on R&D benefits across subsidiaries increases by less than the tax rate for the deduction of R&D expenditures. As a result, a corporate tax increase can increase local R&D of foreign subsidiaries. This cost shifting is a tax efficient reaction if, as it is assumed in the model, the subsidiary that increases its R&D costs reports positive taxable profits, which implies that transfer pricing strategies and other strategies of tax avoidance are insufficient to wipe out all taxable profits. Thus, in our study, we propound the possibly counter-intuitive idea that a higher statutory corporate income tax rate may have positive effects on the local R&D expenditures by MNEs.

In several countries, intellectual property (IP) box regimes provide significantly lower corporate tax rates for income generated from relevant forms of intellectual property and are categorized as income-based tax incentives for R&D and innovation (Bornemann et al., 2018; Alstadsaeter et al., 2018). IP regimes prefer income from patents, but may extend to designs, models, trademarks, copyrights (including software), and certain other categories of intangibles. Some jurisdictions place limitations on where R&D is performed or on the kinds of businesses that qualify for income-based tax relief, like R&D centers (Appelt et al., 2016).

The positive effect of the corporate tax on R&D expenditures may even be amplified by the existence of an IP regime. In this case, if the R&D in the tax-reform country increases, not only the profitability gains of affiliated subsidiaries in other countries are sheltered from the corporate tax increase. In addition, the IP regime allows to spare from the tax rise qualified IP income in the country that introduces the corporate tax increase.

A priory, the role of IP regime for the way in which the corporate tax rate affects R&D expenditures, however, is not unambiguous. While it is true that a lower IP tax rate leaves more of the benefits of R&D to an innovating firm, the existence of an IP box may also reduce the tax rate applicable when a subsidiary deducts its R&D expenditures. *De jure*, most international IP regimes are following the net income approach. In a net income approach, the preferential IP rate applies not only to the revenues earned on the IP. It should also be applied when deducting the cost incurred to produce the IP. If this approach is followed through, an increase in the headline corporate tax rate may not increase the value of the cost deductibility of R&D expenditures and their overall size. In practice, however, it should be difficult to tell apart all those cost from normal business expenses. In this case, for tax purposes, firms have a strong incentive to declare that facilities, and most of the personnel are used for non-R&D-related purposes.

A further reason why an IP box may not prevent that R&D expenditures are deducted against the standard corporate tax rate changes is the application of the gross income approach: a few countries officially allow all R&D costs to be deductible at the higher standard rate, while IP revenues still benefit from the preferential IP rate.

In the end, the question of whether an increase in the corporate tax rate has a higher or lower R&D effect in countries with an IP box is an empirical one. Our empirical analysis of R&D by U.S.-owned MNEs shows that the headline corporate tax has a positive, though insignificant, effect on R&D if there is no IP regime. The effect of a corporate tax increase on R&D expenditures is larger and significant in IP regime countries. This is compatible with the expectation that tax incentives are active, and a large share of R&D deductions for tax purposes is channeled into the standard basket of deductions. We find no significant differences of corporate tax changes depending on whether an IP regime uses the gross or a net income approach.

The empirical result that a higher corporate tax rate tends to have a positive effect on R&D expenditures may not only come as a surprise to many policymakers, but, as to our best knowledge, is new to the literature on taxes and R&D.

When it comes to the effect of an IP regime, we find that given an IP regime is in place, a lower preferential rate on IP income significantly increases R&D expenditures. This suggests a positive effect of the tax preference on the attractiveness for R&D, while Alstadsæter et al., (2018, p. 165) found that, in their sample, the size of the tax advantage of patent boxes led to a surprising negative effect on the probability of moving inventors to the patent box country. At the same time, in our study, the introduction of an IP regime has only a small effect on R&D.

The remainder of the paper is as follows. Section 2 provides a literature review of previous studies that look at how taxes affect the international location of patents. Section 3 introduces a simple model of an MNE’s R&D decisions. Section 4 presents our data, and Sect. 5 discusses the empirical results derived from U.S. MNE expenditures. Section 6 concludes.

## Literature review

Evers et al. (2015) and Bradley et al. (2015) review several objectives that may motivate governments’ decisions to introduce IP regimes.^2^ Both papers suggest that governments that introduce IP regimes aim to reduce tax base erosion, which occurs when IP is shifted to tax havens or other tax law jurisdictions.

Against the background of our own study, the main interest is in existing papers that evaluate the link between taxes, IP regimes, and the amount and location of R&D output.

Most prior studies have measured R&D output in terms of patents and suggest that low and preferential tax rates on IP income lead to more local R&D output. At the same time, the tax-induced increase in patent applications seems associated with a significant increase of the share of patents whose inventors are located abroad. This leaves open whether an IP regime is able to attract also the underlying R&D activity.

Ernst and Spengel (2011) estimate that a decrease of the corporate income tax rate increases the average count of patent applications, the effect being 120% larger for inventions developed by foreign inventors. Griffith et al. (2011) and (2014) confirm that lowering a country’s corporate tax increases the probability that a patent is registered for a firm in that jurisdiction. In addition, Griffith et al. (2011) document that the introduction of IP regimes in Benelux countries increased newly created patents in Benelux countries, but a fall elsewhere. Bradley et al., (2015) suggest a roughly 3% increase in new patent applications for every one percentage point decrease in the tax rate on patent income. Unlike in Griffith et al. (2011), this effect appears to be confined to patents for which the inventors and patent owners are located in the same host country; there seems to be no measurable impact on the number of patents owned and invented in different countries. Evers et al. (2015), Klemens (2016) and Liberini et al. (2018) provide further evidence that preferential tax rate regimes on IP income distort patent registration and lure income on intellectual property to countries that, apart from the IP regimes, are not necessarily perceived as low-tax countries.

Schwab and Todtenhaupt (2017) and Gaessler et al., 2018 investigate the role of restrictions on preferential regimes, with a particular focus on the 'modified nexus approach.' IP boxes with nexus requirement effectively preclude tax benefits from the transfer of intangibles and, thus, seem to result in much smaller cross-border spillovers. Using data on patent applications for a large number of MNEs, Schwab and Todtenhaupt (2021) show that MNE affiliates in non-patent box countries increase their patent activity once the MNE has access to a foreign patent box regime that does not require nexus. Patent boxes that require MNEs to relocate IP and R&D activity (nexus patent boxes) exhibit no significant cross-border effect on average.

While most studies concentrate on the location of new patents, Alstadsæter et al. (2018) not only look at patent registration, but also on the location of researchers. Using patent applications to the EPO of world corporate R&D investors from 39 home countries in 33 different host countries over 2000–12, this paper suggests that patent boxes have a strong effect on patent registrations, especially when these regimes are generous and have a large coverage in terms of the types of IP covered. When it comes to real activity, the tax advantage linked to IP boxes is associated negatively with the annual growth in the number of inventors and also negatively with the probability that a MNE moves inventors from other affiliates to an affiliate in a patent box country.

As intellectual property is firm-specific in nature, arm’s length prices are difficult to obtain. This creates opportunities for MNEs to shift income to low-tax countries by mispricing intra-firm royalties and license fees. Papers looking for evidence on such profit shifting are part of a closely related strand of the literature. Dischinger and Riedel (2011) find that the lower a subsidiary’s corporate tax rate compared to all other affiliates of the same multinational group, including also the parent, the higher is its probability of holding intangible assets there. Karkinsky and Riedel (2012) show that the number of patent applications filed by multinational affiliates strongly responds to changes in corporate tax rate. The estimated semi-elasticity ranges between − 3.5% and − 3.8%. At the same time, there are no statistically significant negative effects on patent applications for purely domestic firms, which lack low-tax affiliates. Bӧhm et al*.* (2012) analyze the extent to which corporations use patents to transfer corporate income to tax favored locations within multinational groups. They provide evidence that low-tax countries are more likely to attract ownership of foreign-invented patents. Indeed, the majority of patents owned in tax-haven locations is invented in a foreign country. Griffith et al. (2014) suggests a negative and statistically significant marginal impact of tax on the payoff from placing legal ownership of a patent in a location, where the own-tax semi-elasticity of patent location choice varies between –0.5% and 3.9%. Dudar and Vogel (2016) conclude that companies seem to use intangible assets as an instrument of base erosion and profit shifting. Several other studies provide more direct evidence on the fact that IP ownership creates opportunities for strategic mispricing of intrafirm trade (e.g., Hebous & Johannesen, 2021; Hopland et al., 2018; Liu et al., 2017). Recently, Baumann et al. (2020) provide descriptive evidence on the negative correlation between a country’s patent income tax rate and its fraction of foreign-invented patents, suggesting that the propensity to locate patent ownership in foreign tax haven economies increases in the inventor country’s patent income tax rate.

A rich study on the tax effects on innovation and patents in the U.S. is Akcigit et al. (2022). While it shows that the corporate tax has a significantly negative effect on patents, this comes “predominantly from mobility responses” (p. 332) suggesting that aggregate effects across U.S. are zero-sum. National tax rate differences are also the analyzed in Lichter et al. (2021) who are analyzing negative tax effects on R&D in Germany. This leaves open the effect of international tax rate differences. Knoll et al. (2021) consider multinational firms and their reactions to input-related R&D tax incentives such as tax credits, accelerated depreciation or super-deductions. The results suggest that MNEs respond to R&D tax incentives by relocating patent activity within the MNE rather than by increasing their aggregate patent activity.

## A model of R&D location within an MNE

This section studies the decision making on R&D expenditures in a simple model, in which an MNE consists of two subsidiaries in two different countries, labeled 1, 2.^3^ The main objective of the exercise is to show that the size of the standard corporate tax rate may have a positive effect on local R&D if (i) R&D cost are deductible against this standard rate and (ii) the benefits of R&D are partly taxed at some other rate, either at a foreign one or at a rate deriving from a preferential IP regime.

In our framework, R&D expenditures of one subsidiary increase the productivity of both subsidiaries. A further effect of R&D and the production of IP is that it leads to royalties for the subsidiary that carries out the respective R&D, leading to an extra benefit for the R&D-conducting subsidiary. We distinguish two tax rates in both countries. ${t}_{i}$ denotes the standard corporate tax rate in country $i=\mathrm{1,2}$; ${\tau }_{IPi}$ is the rate on royalty income in country $i$, that may benefit from an IP regime, such that this rate may or may not fall short of ${t}_{i}$.

The IP, labeled $P$, within the MNE derives from the sum of IP of both subsidiaries, ${P=P}_{1}+{P}_{2}$. Each subsidiary produces IP using a strictly concave research function, such that ${P}_{i}={P}_{i}\left({R}_{i}\right),\frac{\partial {P}_{i}}{{\partial R}_{i}}>0, \frac{{\partial }^{2}{P}_{i}}{{{\partial R}_{i}}^{2}}<0$ where ${R}_{i}$ denotes the research expenditures of subsidiary $i$. As various countries are using expenditure-based R&D incentives we introduce the variables ${\gamma }_{1},{\gamma }_{2}\le 1$ which measure the private cost R&D net of possibly expenditure-based subsidies. Assuming otherwise identical cost of research across countries, ${R}_{i}$ is a measure of the amount of research undertaken in country $i$. The profit in each subsidiary is then a strictly concave function of total IP, ${f}_{i}\left(P\right)$. Royalty incomes for the subsidiaries in country 1 and 2 are simply modeled as ${sP}_{1}\left({R}_{1}\right)$ and ${sP}_{2}\left({R}_{2}\right)$, where $s$ may be thought of as the share of IP that not only can be used to increase MNE productivity but can also be sold on the market. An alternative way of modeling the returns of R&D would assume intra-company payments of royalties. Quantitative evidence on the share of IP-related trade that occurs within MNEs is scarce, but evidence in Hebous and Johannesen (2021, p. 7) for trade of German tax haven affiliates suggests that the majority of trade is with third parties. Net of tax, the global MNE profit derives as:

1 $$\Pi =\left(1-{t}_{1}\right)\left\{{f}_{1}\left(P\right)-{\gamma }_{1}{R}_{1}\right\}+\left(1-{\tau }_{IP1}\right)\left\{s{P}_{1}\left({R}_{1}\right)\right\} +\left(1-{t}_{2}\right)\left\{{f}_{2}\left(P\right)-{{\gamma }_{2}R}_{2}\right\}+\left(1-{\tau }_{IP2}\right)\left\{s{P}_{2}({R}_{2})\right\}.$$

Here, we assume that intramarginal profits of affiliates are always sufficient to fully deduct R&D cost. The Lagrangian of the profit maximization problem can be written as $L=\Pi +{\mu }_{1}{R}_{1}+{\mu }_{2}{R}_{2}$, where ${\mu }_{1}$ and ${\mu }_{2}$ represent Kuhn–Tucker multipliers for the non-negativity constraints on local research expenditures. The two first-order conditions for a profit maximum are:

2 $$\left( {1 - t_{i}} \right)\left\{{\frac{{\partial f_{i}}}{{\partial P}}\frac{{\partial P_{i}}}{{\partial R_{i}}} - \gamma_{1}} \right\} + \left( {1 - \tau_{IPi}} \right)s\frac{{\partial P_{i}}}{{\partial R_{i}}} + \left( {1 - t_{j}} \right)\left\{ {\frac{{\partial f_{j}}}{{\partial P}}\frac{{\partial P_{i}}}{{\partial R_{i} }}} \right\} + \mu_{i} = 0\;\;for\;i,j = 1,2;\;i \ne j$$

We consider a special case, which is particularly easy to analyze, where just one subsidiary, say in country 1, conducts research $\left({\mu }_{1}=0;\;{R}_{1}>0\right)$ and the other subsidiary is in a corner solution with $({\mu }_{2}>0;\;{R}_{2}=0)$. This reflects that MNEs typically concentrate research in few locations. In this case, a marginal change ${\text{d}}{t}_{i}$ or ${{\text{d}}\tau }_{IP1}$ leaves constant ${R}_{2}$.

A change in the corporate tax rate of country 1, may have different effects, depending on whether an IP regime is in place or not. The effect of a corporate tax rate change in the presence of an IP regime can be derived by marginally changing ${t}_{1}$, but leaving the rate on IP income (royalties) constant (${{\text{d}}\tau }_{IP1}=0)$. By total differentiation of the first-order condition we receive for ${\text{d}}{R}_{2}=0$:

3 $$\frac{{\text{d}}{R}_{1}}{{\text{d}}{t}_{1}}=\frac{\frac{\partial {f}_{1}}{\partial {P}}\frac{\partial {P}_{1}}{\partial {R}_{1}} -{\upgamma }_{1}}{\left(1-{\tau }_{IP1}\right)s\frac{{\partial }^{2}{P}_{1}}{\partial {{R}_{1}}^{2}}+A\left(1-{t}_{1}\right)+B\left(1-{t}_{2}\right)}>0$$

where

$$A\equiv \frac{{\partial }^{2}{f}_{1}}{\partial {P}^{2}} {\left(\frac{\partial {P}_{1}}{\partial {R}_{1}}\right)}^{2}+\frac{\partial {f}_{1}}{\partial {P}}\frac{{\partial }^{2}{P}_{1}}{\partial {{R}_{1}}^{2}}<0;B\equiv \frac{{\partial }^{2}{f}_{2}}{\partial {P}^{2}} {\left(\frac{\partial {P}_{1}}{\partial {R}_{1}}\right)}^{2}+\frac{\partial {f}_{2}}{\partial {P}}\frac{{\partial }^{2}{P}_{1}}{\partial {{R}_{1}}^{2}}<0$$

The positive sign of $\frac{{\text{d}}{R}_{1}}{{\text{d}}{t}_{1}}$ in Eq. (3) results as, due to our concavity assumptions, all three terms in the denominator are negative and, at the same time, the numerator is negative from the first-order condition. It establishes a somewhat counter-intuitive result according to which a corporate tax increase can positively affect local R&D.

If country 1 changes its corporate tax rate without having an IP regime in place, the effect on ${R}_{1}$ needs modification as ${\text{d}}{t}_{1}={\text{d}}{\tau }_{IP1}$ and the effect of a corporate tax increase derives as:

4 $${\left.\frac{{\text{d}}{R}_{1}}{{\text{d}}{t}_{1}}\right|}_{{\text{d}}{t}_{1}={\text{d}}{\tau }_{IP1}}=\frac{\frac{\partial {f}_{1}}{\partial {P}}\frac{\partial {P}_{1}}{\partial {R}_{1}} -{\upgamma }_{1}+s\frac{\partial {P}_{1}}{\partial {R}_{1}}}{\left(1-{\tau }_{IP1}\right)s\frac{{\partial }^{2}{P}_{1}}{\partial {{R}_{1}}^{2}}+A\left(1-{t}_{1}\right)+B\left(1-{t}_{2}\right)}>0$$

With ${t}_{1}={\tau }_{IP1}$, the numerator (as the denominator) continues to be negative, but the positive term $s\frac{\partial {P}_{1}}{\partial {R}_{1}}$ tends to dampen the positive effect of an increase in ${t}_{1}$. Evaluating Eqs. (3) and (4) for the same set of initial tax rates yields $\frac{{\text{d}}{R}_{1}}{{\text{d}}{t}_{1}}>{\left.\frac{d{R}_{1}}{d{t}_{1}}\right|}_{d{t}_{1}=d{\tau }_{IP1}}>0.$ If the tax rate increases in country 1, the value of tax-deductibility of R&D there is more valuable in both cases. At the same time, at least some benefit of R&D does not fall under the higher tax rate. In the case, in which there is no IP box in country 1, this is the case since benefits of R&D arise in country 2 and are taxed there. If country 1 employs an IP box, royalties received from third parties are also sheltered from the higher tax, which is the intuition behind the first inequality.

Next, consider a marginal change in ${\tau }_{IP1}$ assuming an IP regime is present and ${\text{d}}{t}_{1}=0.$ From differentiating the first-order condition, we now get.

5 $$\frac{{\text{d}}{R}_{1}}{{\text{d}}{\tau }_{IP1}}=\frac{ s\frac{\partial {P}_{1}}{\partial {R}_{1}}}{\left(1-{\tau }_{IP1}\right)s\frac{{\partial }^{2}{P}_{1}}{\partial {{R}_{1}}^{2}}+A\left(1-{t}_{1}\right)+B\left(1-{t}_{2}\right)}<0$$

This leads to the formulation of the following hypotheses, which are empirically testable.

### H1.

An increase in the corporate tax rate of a country increases the local R&D expenditures of MNEs.

### H2.

The increase in local R&D expenditures of MNEs upon an increase in the corporate tax rate is higher if IP income in the respective country is subject to a separate tax rate (IP regime) than if this is not the case.

### H3.

If there is an IP regime in place, an increase of the local tax rate on IP income will decrease local R&D.

Clearly, the above model introduced a very simple framework. Several limitations come to mind and may or may not be important factors in practice.

The corporate tax modeled above resembles a pure profits tax, as all costs are tax deductible. Real world corporate taxes differ and usually disallow deduction of some cost, for example, the opportunity cost of equity. Therefore, high real-world taxes, unlike the corporate tax in the above model, may induce the MNE to exit a country or enter a different one. This would have a negative effect on R&D in high-tax countries, possibly negatively affecting the empirical support for H1 and H2. Despite this possibility, the mechanism described above would work against such a reduction of R&D and cushion the effect of a corporate tax increase.

Another caveat applies to the implicit assumption of the model that tax rates indeed are relevant as all subsidiaries have positive taxable profits. In so far, as some real-world MNEs have sufficiently powerful tax-avoidance instruments that already wipe out taxes, the above mechanisms would have reduced predictive power.

Another possible concern is that the model does not explicitly allow for contract R&D. Within an MNE, a low-tax subsidiary could pay a high-tax subsidiary to conduct R&D services on behalf of the low-tax subsidiary (Griffith et al., 2014, p.14). While this has not been explicitly modeled, the possibility of such schemes should reinforce the expectation that the cost deductibility of R&D expenditures is an argument to conduct real R&D activity in high-tax countries, given that the MNE wishes to be present in those countries.

## Data

The empirical part of the paper uses aggregated data, although the above model discussed the decision problem of a single MNE. Unfortunately, company accounts data does not typically distinguish the geographical location of firm’s R&D activities and multinationals report R&D expenditures at consolidated level. Therefore, we use data on R&D expenditures of majority-owned foreign affiliates of U.S. MNEs, reported at country level. We obtain the data from the Bureau of Economic Analysis homepage (BEA, 2020)*.*

The BEA database contains the R&D expenditures of U.S. majority-owned foreign affiliates as performed by the relevant foreign affiliates. Should one affiliate pay a second affiliate within the same MNE to conduct R&D, then the R&D expenditure would be attributed to that second affiliate; no R&D cost are recorded for the first, merely contracting U.S. affiliate.^4^ This accounting convention is adequate for our purpose, as our main interest is in where the actual research activities take place and how these activities are affected by taxation.^5^

Our sample includes up to seventy-five countries where U.S. multinationals have reported R&D expenditures for at least some years during the period under analysis, 2009–2017.^6^ In total, the sample includes 621 country-year observations, resulting in a slightly unbalanced panel with only 54 missing country-year entries.

The presence of an IP regime in the host country in a certain year constitutes the first variable of interest in our empirical analysis. Table 5 in Appendix A reports information on the existence of an IP regime in each country-year, the year of enactment and the preferential tax rate on IP income. For the construction of this variable, we rely on OECD (2015, 2017). Further sources used for identifying IP regimes where data from multinational professional services networks such KPMG, PwC, Deloitte, as well as from national websites, and previous academic papers.

A second variable of main interest is the tax rate on IP income. As in the model of Sect. 3, it equals the preferential IP tax rate for countries that run an IP regime. For countries that do not run an IP regime, the tax rate on IP income equals the statutory corporate income tax rate. We take the information on the statutory corporate income tax rate from the OECD Statistics Database, KPMG, and Eurostat. For the preferential corporate income tax rates on IP income, the sources of information are significantly broader and coincide with the ones used for data collection on the presence of IP regimes in each country.^7^

Finally, we add control variables on tax and non-tax country characteristics. First, we control for the presence of input-related tax incentives per country by introducing a dummy variable, expenditure-based tax incentives (EBTI), which equals one if country *c* in year *t* offers at least one of the four R&D-related tax incentives, namely tax credits, tax allowance, accelerated depreciation and/or super deductions, and 0 otherwise. Qualitative information for the quantitative construction of the EBTI dummy variable is obtained from Ernst and Young Worldwide R&D incentives reference guides, PwC Worldwide Tax Summaries, OECD R&D Tax Incentives database, KPMG’s Europe, Middle East & Africa region (EMEA) research and development (R&D) incentives guide, as well as national websites. We obtain this information for 68 out of 75 countries in our original sample.^8^

We refer to prior literature (Bӧhm et al*.,* 2012; Dudar & Voget, 2016; Karkinsky & Riedel, 2012; Alstadsaeter et al*.,* 2018; Baumann et al., 2020; Mukherjee et al., 2017; Becker et al., 2020), for the choice of the control variables in the baseline regression and for the two additional control variables used in the robustness checks analysis. We include LN (GDP) to control for market size, which measures the log of GDP in purchasing power parities. Since tax rates and country sizes have been found to be systematically correlated, inclusion of this size measure prevents the tax rate from picking up size effects (Ruf & Weichenrieder, 2012; Weichenrieder, 2005). In order to control for the country’s degree of development and living standards, the logarithm of GDP per capita is included. In line with Dischinger and Riedel (2011), as a proxy for the country’s economic situation, we include the unemployment rate. The corruption perception index (CPI) represents the transparency International corruption index, which is constructed with higher values of the index indicating lower corruption, in order to capture perceptions of the public sector corruption, the quality of public services, the quality of policy formulation and implementation, and the credibility of the governments’ commitment to such policies. Table 3 in Appendix A reports summary statistics of all variables used. As a robustness check (Appendix B, Table 6), we also included a Property Rights Index as included in several studies (Alstadsæter et al., 2018; Becker et al., 2020; Bradley et al., 2015; Griffith et al., 2014; Karkinsky & Riedel, 2012) and trade openness (Trade) as in Ernst and Spengel (2011). We excluded these consistently insignificant variables in the regressions of Table 1.

**Fig. 1:**
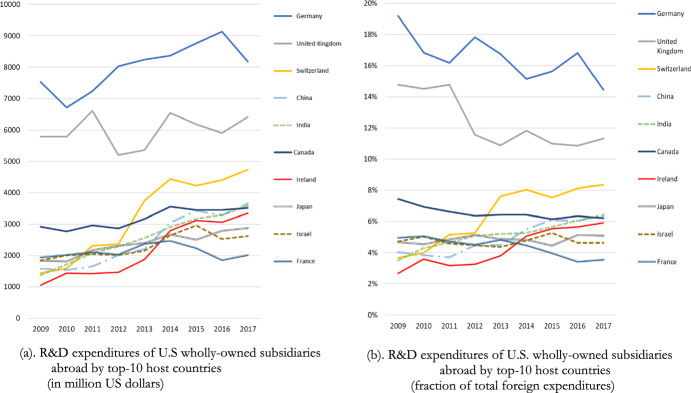
**a** R&D expenditures of U.S wholly-owned subsidiaries abroad by top-10 host countries (in million US dollars) **b** R&D expenditures of U.S. wholly-owned subsidiaries abroad by top-10 host countries (fraction of total foreign expenditures) Source: Bureau of Economic Analysis (BEA)

Figure 1 visualizes the R&D expenditures in the top-10 host countries in absolute and relative terms. Germany, a high-tax country, leads the top-10 countries where U.S. multinationals locate their R&D expenditures, accounting for between 6700 and 9200 million dollar or between 14 and 20% of total foreign R&D expenditures. Germany is followed by United Kingdom and Canada until 2012 (Switzerland after 2012). Among the top-10 countries, four of them, Germany, Canada, Switzerland^9^ and Japan, fail to have a preferential tax rate regime during the period under consideration. The first three of them lead the top-10 list. The statutory corporate tax rate in Japan is the highest among all countries where U.S. majority-owned subsidiaries invest in R&D, with a range between 30.89% in 2017 and 40.69% in 2009. India as well offered one of the highest statutory corporate income tax rates hovering around 34% and 35% between 2009 and 2017, but it implemented an IP box regime in 2016 with a preferential tax rate on IP income of about 10%. China, that enacted an IP regime in 2008 and France, which has an IP regime since 1971, have the highest preferential tax rate (15%) among the rest of the six countries that run an IP regime. The United Kingdom, after the 2013 enactment of an IP box introduced a preferential tax rate of 10%. In the year of IP box enactment, the tax advantage was about 13%. It decreased to 9% after a year-by-year decrease of the statutory corporate income tax rate (19% in 2017).

A country with frequent changes is Israel. Its statutory corporate income tax rate ranges between 24 and 26% and its preferential tax rate within the period changes from 10 to 7%, then again to 9% and in the last two years 2016, 2017 it decreases to 6%. Ireland, which provided the lowest tax rate on IP income, 2.5% for 2009 and 2010, abolished its IP regime in 2010 and re-introduced it in 2016 with a preferential tax rate of 6.5%.

**Fig. 2:**
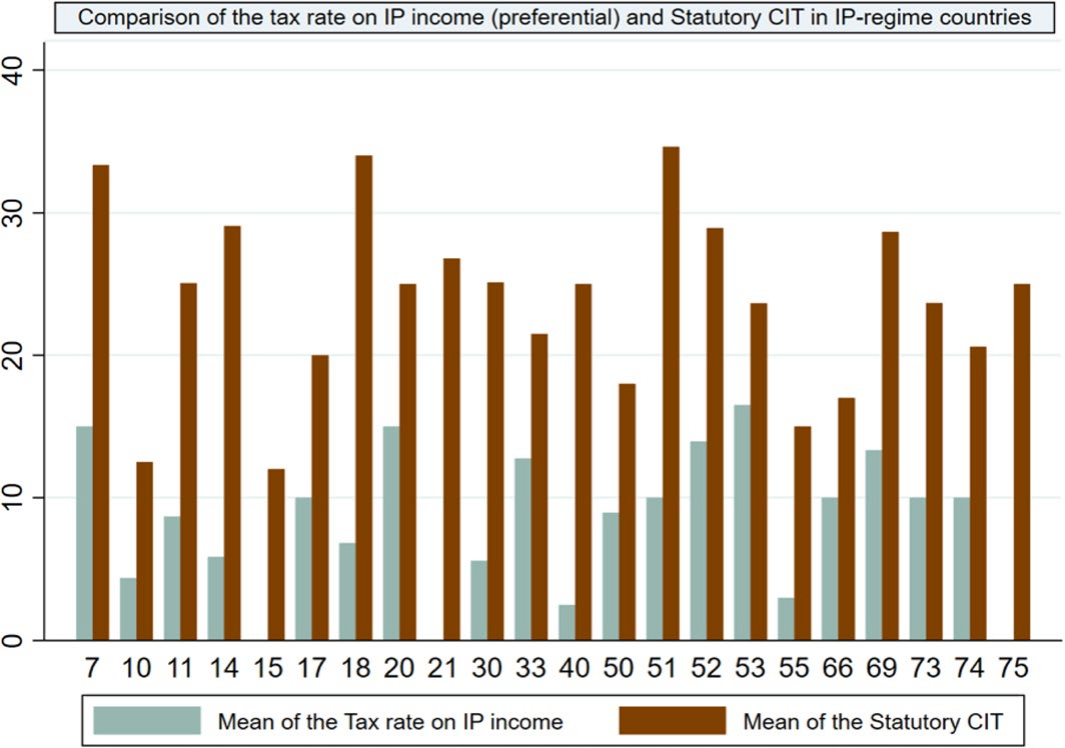
Comparing the preferential tax rate on IP income and statutory corporate income tax rate in IP-regime countries. Note: Countries corresponding to the country codes in our sample, as shown in the horizontal axis: 7-France; 10-Ireland; 11-Israel; 14-Luxembourg; 15-Macau; 17-Turkey; 18-Belgium; 20-China; 21-Colombia; 30-Netherlands; 33-Portugal; 40-Barbados; 50-Hungary; 51-India; 52-Italy; 53-Korea, Republic of; 55-Mauritius; 66-Singapore; 69-Spain; 73-Thailand; 74-United Kingdom; 75-Uruguay

Figure 2 for each of the 22 countries with IP regimes, compares the mean corporate tax rate and the mean preferential IP rate across the years when an IP-regime was in place. The largest difference between the two mean rates is in Uruguay, Colombia and Macau, the smallest difference applies to the Republic of Korea.

Figure 1 R&D expenditures of U.S. wholly-owned subsidiaries by top-10 host countries in (a) millions of dollars and (b) as a fraction of total foreign R&D expenditures.

Keen (2001) and others have argued that preferential tax regimes may allow for higher standard corporate tax rates since, with such a special regime, parts of the most mobile tax base are taken out of the high-taxed base. We checked whether this is reflected in our sample. Twelve out of 22 IP-regime countries had the regime in place during all of our sample years; 10 countries had years with and without IP regimes. For these 10 countries, the mean statutory corporate tax rate in years without an IP regime (24.6%) is almost identical to the average rate in years with an IP regime (23.2%). Controlling for country and time-fixed effects, we found an insignificant negative correlation between an IP regime dummy and the rate of the statutory corporate tax rate. This finding does not support the idea that countries introduce a preferential regime to be able to increase their rates on the remaining tax base.

Among the 22 countries, Ireland, Israel, Netherlands, Portugal, Hungary, Italy, Korea, and Spain had different rates of the preferential tax rate on IP across the years in which a regime had been in place. For the rest, the tax rate on IP income did not change.

## Estimations

In this section, we exploit our panel data to regress country-level R&D expenditures of US-owned subsidiaries on country characteristics that capture R&D-friendliness from a tax and non-tax perspective. Based on the model in Sect. 3, our main interest is in the corporate tax rate, the availability of a preferred patent box regime, and the interaction between tax rates and regimes. In addition, we control for the availability of expenditure-based R&D incentives. This leads us to the following empirical model.

6 $${\text{LN}}\left( {{\text{R}}\& {\text{D}}} \right)_{{{\text{it}}}} = \alpha_{0 } + \, \beta_{{{1} }} {\text{ipr}}_{{{\text{it}}}} + \, \beta_{{2}} \tau_{{{\text{IP}}\_{\text{it}}}} + \beta_{{3}} {\text{Stat}}\_{\text{CIT}}_{{{\text{it}}}} + \delta_{{1}} {\text{Stat}}\_{\text{CIT}}_{{{\text{it}} }} *{\text{ ipr}}_{{{\text{it}}}} + \, \beta_{{{4} }} {\text{X}}_{{{\text{it}}}} + \varphi_{{{\text{i}} }} + {\text{ E}}_{{{\text{it}}}} + \, \gamma_{{{\text{t}} }} + {\text{u}}_{{{\text{it}}}}$$

Our left-hand variable, LN (R&D)_it_, represents the natural log of R&D expenditures by U.S. majority-owned subsidiaries in country *i* in year *t*.^10^ In each country, there are two, possibly distinct, tax rates: the standard statutory corporate income tax rate applying to all kinds of deductions and sales revenues (Stat_CIT_it_), and the tax rate as it applies specifically to income from intellectual property (τ_IP_it_). Although these rates may be identical if the country under consideration does not offer a patent box regime, this separation allows to account for regime changes over time and a lower rate on income from intellectual property. The dummy variable ipr_it_ takes on the value one if in country *i* in year *t* there is an IP regime in place, and zero otherwise. The interaction term Stat_CIT_it_ * ipr_it_ captures the possibly different effect of the statutory corporate income tax rate on R&D expenditures if an IP box is in place. In this case, an increase in the corporate tax may increase the value of the tax deductibility of research expenditures but would ceteris paribus not increase the tax on IP income. For this reason, we expect a positive coefficient for this interaction that captures a difference in the marginal effect of the statutory corporate income tax rate between countries with an IP-regime in place and countries that do not offer a preferential tax treatment for IP income. *E*_it_ captures the existence of expenditure-based R&D incentives, *X*_it_ is a vector including the four country-specific control variables, described in the previous section. The variable *φ*_i_ represents country-fixed effects, capturing a country’s unobserved characteristics that are time-invariant.^11^ The variable γ_t_ captures year-fixed effects that may affect all host countries alike.

While OLS is easy to interpret, a logarithmic or semi logarithmic OLS model may be biased if the data is heteroscedastic (Silva and Tenreyo, 2006). Another possible concern is that OLS does not account for the fact that the dependent variable is restricted to positive values (Alstadsæter et al., 2018; Karkinsky & Riedel, 2012). Moreover, country-years with zero R&D are dropped by taking the log. In order to account for these concerns, we also use a negative binomial (NB) fixed effects model. We follow Allison and Waterman (2002) and Greene (2004), suggesting a simple approach which jointly estimates the parameters, fixed effects and the over-dispersion model in a standard NB model with a full set of country specific dummy variables.^12^

A further potential issue are endogeneity problems. For example, countries could be tempted to introduce an IP regime in years in which R&D expenditures of local U.S. are particularly low. This produced a problem of reverse causality. This is a general problem of the IP regime literature, which according to our knowledge has not been addressed fully convincingly in previous cross-country studies. See the discussion in Alstadsæter et al., (2018, p. 150). This said, we want to note that a main interest of this study is to evaluate the role of the general corporate tax rate on R&D. Compared to the introduction of IP regimes, this general rate should be less susceptible of being set as an intentional instrument of R&D policy. If true, this should reduce the problem of endogeneity compared to the papers on IP regimes reviewed in Sect. 2. A potential omitted variables problem is addressed by using country fixed effects and further time-varying country characteristics.

### Empirical results

Columns (1) and (2) in Table 1 report on OLS fixed effects model estimations, columns (3) and (4) report on the NB model. The NB estimations contain the same set of regressors as the OLS regression in column (1) and column (2). While the left-hand side is now measured in level rather than its logarithm, the coefficients of tax rates in the negative binomial model can be interpreted as semi-elasticities. To account for heteroscedasticity and possible serial correlations, we cluster all estimates in Table 1 at the country level.^13^

**Table 1:** Estimating tax effects on international R&D expenditures of U.S. majority-owned subsidiaries

Dependent variable: R&D Expenditures of U.S. majority-owned subsidiaries				
Model	OLS	OLS	NBM	NBM
	LN (R&D Expn.)	LN (R&D Expn.)	R&D Expn	R&D Expn
Regressors	(1)	(2)	(3)	(4)
IP Regime (dummy) (ipr_it_)	− 0.107 (0.215)	− 0.276 (0.216)	0.0750 (0.177)	− 0.140 (0.185)
Tax rate on IP income (τ_IP_it_)	− 0.0264** (0.0102)	− 0.0244** (0.0106)	− 0.0262** (0.0118)	− 0.0239** (0.0114)
Statutory CIT (Stat_CIT_it_)	0.0379** (0.0143)	0.0358** (0.0137)	0.0474*** (0.0148)	0.0401*** (0.0142)
IP Regime (dummy) * Statutory CIT (ipr_it_ * Stat_CIT_it_)	− 0.0153 (0.0124)	− 0.00832 (0.0120)	− 0.0190 (0.0125)	− 0.0101 (0.0117)
Expenditure-based tax incentives (EBTI)	− 0.289 (0.256)	− 0.364 (0.262)	− 0.155 (0.194)	− 0.179 (0.190)
LN (GDP)		0.643* (0.328)		0.611** (0.276)
LN (GDP pC)		0.0179 (0.379)		0.0433 (0.291)
Unemployment		− 0.0313* (0.0169)		− 0.0303** (0.0150)
Corruption Perception Index (CPI)		0.00402 (0.00313)		0.00192 (0.00329)
(i) τ_IP_it_ + Stat_CIT_i_	0.0115 (0.234)	0.0115 (0.205)	0.0212** (0.031)	0.0163* (0.094)
(ii) (1 + ipr_it_)*Stat_CIT_it_	0.0225** (0.014)	0.0274*** (0.007)	0.0283*** (0.000)	0.0300*** (0.000)
(iii): (ii)–(i)	0.0111 (0.119)	0.0160** (0.040)	0.0071 (0.187)	0.0138** (0.027)
Obs	547	531	570	546
Nr. of countries	68	65	68	65
*R*^2^ (within)/Pseudo *R*^2^	0.37	0.38	0.30	0.30
Log pseudolikelihood			− 2714.06	− 2627.20
Alpha for overdispersion (std. error)			(0.0665) (0.0137)	(0.0539) (0.0117)

The dependent variable in columns (1) and (2) is the log of R&D expenditures, in columns (3) and (4) R&D expenditures (in $mill). Levels of significance: ***p < 0.01, **p < 0.05, *p < 0.1. p-values are based on robust standard errors, clustered at the country level. Countries are observed during 2009–2017 (unbalanced sample). All estimations include country-fixed effects and time-fixed effects. The unit of observation is country-year. The alpha parameter informs about the degree of dispersion, if alpha is significantly greater than zero the data are over dispersed and are better estimated using a negative binomial model than a Poisson model. The negative binomial model uses country dummies, which unlike a conditional negative binomial model controls for *all* invariant covariates

We first focus on the interpretation of column (2), where the full set of control variables in an OLS regression is included to discuss the hypotheses H1–H3 of Sect. 3. As discussed in that section, a change in the statutory corporate tax rate may be different depending on whether the variation happens with an IP regime in place or not.

In the absence of an IP regime, ${ipr}_{it}=0$. In this case, taking the derivative of Eq. (6), we have $\frac{\mathrm{d}LN(R\&D)}{\mathrm{d}{Stat\_CIT}_{it}}={\beta }_{2}+{\beta }_{3}.$ In the absence of an IP regime, a change in Stat_CIT, by construction, goes along with a change in τ_IP_it_, as the standard corporate tax then also applies to IP income. This means that, in this case, a one percentage point increase in the CIT leads to an increase in R&D expenditures by some 1.2% (= − 0.0244 + 0.0358) according to column (2). This, however, is not statistically significant in the OLS estimations, and only marginally statistically significant in the full NB model. Thus, it lends only weak support to H1. Section 3 discusses potential reasons, why effects outside our model could actually lead to a negative effect of the corporate tax rate on R&D. Against this background, it is an interesting observation that a higher tax rate does not seem to have a negative effect on R&D.

Compared to when an IP regime is not in place, H2 expresses the expectation that an increase of the corporate tax has a more positive effect on R&D if such a regime is in place. A change in the corporate tax rate, in this case, leaves the tax on IP income constant as this income is subject to a separate rate. Formally, from Eq. (6), if ${ipr}_{it}=1$  we have $\frac{\mathrm{d} LN(R\&D) }{\mathrm{d Stat}\_\mathrm{CIT}}$= β_3_ + δ_1_.

The marginal effect in column 2 then derives from the addition of the coefficient of Stat_CIT and the coefficient of the interaction effect IP Regime (dummy) * Statutory CIT: (0.0358 – 0.00832 =) 2.74% (cf. line (ii)). This linear combination of coefficients is significantly different from zero at the 1%-level. At the 5%-significance level, the marginal effect of the corporate tax increase with an IP regime in place (2.74%) is higher than the marginal effect without a regime (1.2%) according to line (iii). While significance levels are slightly lower in the NB models for some coefficients, the general pattern is preserved and the size of the coefficients is closely comparable, which should give further credibility to the OLS estimates.

The observation that a marginal change in the corporate tax rate has a larger effect if an IP regime is in place is in line with our expectation (H2). In countries without a preferential tax rate regime on IP income, a higher corporate tax makes the deductibility of R&D cost more valuable, but also increases the tax on R&D returns. This is different in IP regime countries, where the income generated from IP is sheltered by the IP regime.

The positive and strongly significant effect of the statutory CIT on R&D expenditures in these countries is compatible with the view that subsidiaries manage to deduct a large share of the cost of R&D at the higher statutory corporate tax rate, while the returns of R&D investments benefit from the lower IP rate. Although formally this is only allowed under the gross income approach, it could be that countries are lenient under the net income approach and effectively there is always a de facto gross approach in place. With R&D costs largely consisting of labor costs, firms might easily report R&D costs as normal costs in order to get their deductibility under the normal statutory corporate income tax rate, while maintaining returns from R&D taxed at the lower (preferential) corporate income tax rate.

The coefficient of *IP Regime* (dummy) is not statistically significant across columns. The sign of the *IP Regime* (dummy) cannot be taken as evidence that IP regimes are insignificant, as the dummy appears in interactions with two tax rates. Formally, from Eq. (6), we have $\frac{\mathrm{d} LN(R\&D) }{\mathrm{d }ipr }$= β_1_ + β_2_(dτ_IP_ /d$ipr$) + δ_1_ Stat_CIT_it._ Assuming that the introduction of an IP regime reduces the applicable rate from the average of the corporate income tax rate (24.1%) rate to the average rate of IP regimes in the sample (8.0%), from column (2), we receive only a small insignificant overall effect of $-0.086$ ($=-0.276+16\bullet 0.0244-0.00822\bullet 24)$ on the log of R&D expenditures. The significantly negative coefficient of the variable τ_IP _it_ indicates that (given an IP regime is in place) a lower tax rate on IP income (keeping the corporate income tax constant) indeed is associated with higher R&D expenditures: a *reduction* of the rate on IP income by one percentage point increases local R&D expenditure by some 2.4%.^14^ Again, the results are closely comparable across columns and models (OLS/NB), providing support for H3.

Expenditure-based tax incentives (EBTIs) seem to exert no impact on R&D expenditures, which is somehow in line with Knoll et al. (2021). Firms hardly raise their R&D activities due to generous input-related R&D tax incentives. Furthermore, the coefficients of the four control variables have plausible signs. We find a positive effect of country size on R&D, as measured by the coefficient of LN (GDP). Freedom of corruption (CPI) enters positively, although only insignificantly. A negative effect of the unemployment rate is weakly significant in the OLS and statistically significant at the 5% in the NB model. GDP per capita, LN (GDP pC), enters positively, but without statistical significance.

### Gross income approach

The results in Table 1 are based on a pooling of IP regimes with gross and net approaches. This reflects the expectation that, for tax purposes, it is difficult to tell apart R&D related expenditures from other expenditures. In such a situation, MNEs have, for tax purposes, the incentive to flag R&D expenditures as normal expenditures to receive an increased tax shelter and de facto the distinction of the net and gross approach should be of restricted relevance in practice.

At the same time, it is possible to tell apart the few countries that indeed use a gross approach *de jure.* It should be kept in mind, though, that in this case some results are then based on a very limited subsample. In our data set, out of the 22 countries that used an IP regime during 2009–2017, only Belgium, Hungary, Portugal, and Spain used the gross approach of an IP regime at least for some years.^15^ As one of our main interests lies in the R&D effect from a change of the corporate tax rate (i.e., in an interaction effect), identification depends on observing corporate tax rate changes while an IP regime is in place. In the group of the four IP regimes countries with a gross approach, only two, Hungary and Portugal, had at least one corporate tax rate change during our sample period. Three of the four countries had a tax rate change when it comes to the rate on IP income (Hungary, Portugal and Spain).

To identify possibly different effects for gross and net income approaches, we slightly modify our empirical framework. We introduce a further dummy variable named Gross_approach_it_, which takes on the value one if a country with a preferential IP regime in year* t* allows the current R&D expenses to be deducted from non-IP income, which is taxed at the regular corporate tax rate. In addition, this new dummy is interacted with the standard corporate tax rate and the IP rate forming the variables Gross_approach*Stat_CIT and Gross_approach*τ_IP_. Consequently, the new regression equation reads:

7 $$\begin{gathered} {\text{LN }}\left( {{\text{R}}\& {\text{D Expn}}.} \right)_{{{\text{it}}}} = \alpha_{0 } + \, \beta_{{{1} }} {\text{ipr}}_{{{\text{it}}}} \, + \, \beta_{{2}} \tau_{{{\text{IP}}\_{\text{it}}}} + \beta_{{{3} }} {\text{Stat}}\_{\text{CIT}}_{{{\text{it}}}} \hfill \\ + \delta_{{{1} }} {\text{Stat}}\_{\text{CIT}}_{{{\text{it}} }} *{\text{ ipr}}_{{{\text{it}}}} + \beta_{{4}} {\text{Gross}}\_{\text{approach}}_{{{\text{it}}}} + \, \delta_{{2}} {\text{Gross}}\_{\text{approach}}_{{{\text{it}}}} *{\text{ Stat}}\_{\text{CIT}}_{{{\text{it}}}} \hfill \\ + \, \delta_{{3}} {\text{Gross}}\_{\text{approach}}_{{{\text{it}}}} * \, \tau_{{{\text{IP}}\_{\text{it}} }} + \beta_{{5}} {\text{X}}_{{{\text{it}} }} + \varphi_{{{\text{i}} }} + \, \gamma_{{{\text{t}} }} + {\text{ u}}_{{{\text{it}}}} \hfill \\ \end{gathered}$$

Equation (6) allows the corporate tax rate (Stat_CIT_it_) having a different marginal effect depending on whether there is a not or gross approach in place. Taking the derivative of Eq. (7) with respect to Stat_CIT_it_, we receive *β*_3_ + *δ*_1 _+ *δ*_2_ in case of a net approach and *β*_3_ + *δ*_1_ in the case of a gross approach. This means that the differential effect is captured by δ_2_.

Table 2 reports the results for Eq. (7). The four columns again report on OLS and NB regressions. The full set of controls is included in columns (2) and (4), while in columns (1) and (3) we include the main variables of interest and control only for the presence of expenditure-based tax incentives.

**Table 2:** Tax effects on R&D expenditures: Differentiating between gross and net approaches

Dependent Variable: R&D Expenditures of U.S. majority−owned subsidiaries				
Model	OLS	OLS	NBM	NBM
	LN (R&D Expn.)	LN (R&D Expn.)	R&D Expn	R&D Expn
Regressors	(1)	(2)	(3)	(4)
IP Regime *(dummy)*	0.0399 (0.185)	− 0.189 (0.224)	0.173 (0.164)	− 0.0821 (0.190)
Tax rate on IP income	− 0.0341*** (0.00924)	− 0.0308*** (0.0110)	− 0.0338*** (0.0107)	− 0.0297** (0.0116)
Statutory CIT	0.0442*** (0.0143)	0.0416*** (0.0147)	0.0535*** (0.0146)	0.0453*** (0.0150)
IP Regime *(dummy)* * Statutory CIT	− 0.0265** (0.0104)	− 0.0164 (0.0123)	− 0.0284** (0.0113)	− 0.0165 (0.0122)
Gross approach	− 1.014 (0.717)	− 0.555 (0.508)	− 0.654 (0.689)	− 0.373 (0.456)
Gross approach * Statutory CIT	0.0189 (0.0259)	0.00152 (0.0215)	0.00146 (0.0271)	− 0.0101 (0.0179)
Gross approach * Tax rate on IP income	0.0487 (0.0333)	0.0469***(0.0158)	0.0558 (0.0451)	0.0536*** (0.0203)
Expenditure-based Tax Incentives (EBTI)	− 0.293 (0.255)	− 0.368 (0.263)	− 0.159 (0.192)	− 0.181 (0.191)
LN (GDP)		0.634* (0.338)		0.605** (0.281)
LN (GDP pC)		0.181 (0.387)		0.0479 (0.295)
Unemployment		− 0.0317* (0.0172)		− 0.0298** (0.0150)
Corruption Perception Index (CPI)		0.00372 (0.00309)		0.00163 (0.00334)
(i) τ_IP_it_ + Stat_CIT_i_	0.010	0.011	0.020*	0.016
(ii) (1 + ipr_it_)*Stat_CIT_it_	0.018*	0.025**	0.025***	0.029***
(iii): (ii)–(i)	0.008	0.0143*	0.0054	0.013**
(iv) (1 + ipr_it +_ Gross approach)*Stat_CIT_it_	0.037	0.027	0.027	0.019
(v): (iv) – (ii)	0.019	0.002	0.0015	− 0.010
(vi) τ_IP_it_ (1 + Gross approach)	0.015	0.016	0.022	0.024
Obs	547	531	570	546
Nr. of countries	68	65	68	65
*R*^2^ (within)/ Pseudo *R*^2^	0.31	0.38	0.30	0.30
Log pseudolikelihood			− 2712.63	− 2626.34
Alpha for overdispersion (std. error)			(0.0660) (0.0135)	(0.0537) (0.0117)

The dependent variable in columns (1) and (2) is the log of R&D expenditures, in columns (3) and (4) R&D expenditures (in $mill). Levels of significance: ***p < 0.01, **p < 0.05, *p < 0.1. *p* values in parentheses are based on robust standard errors clustered at the country level. The model is estimated via OLS in regressions (1) and (2) and via a negative binomial model in regressions (3) and (4). Countries are observed during the period 2009–2017 (unbalanced sample). All estimations include country-fixed effects and time-fixed effects. The unit of observation is country-year. The alpha parameter informs about the degree of dispersion, if alpha is significantly greater than zero the data are over dispersed and are better estimated using a negative binomial model than a Poisson model

We first concentrate on the results for OLS in column (2). As in Table 1, the preferential tax rate on IP income enters significantly negative for R&D expenditures, resulting in a semi-elasticity of some -3.1%. As we have added an additional interaction of this rate with the gross approach dummy, the value of 3.1% is estimated for net approach regimes. The interaction with the gross approach dummy enters surprisingly with a positive sign that is statistically significant in columns (2) and (4), although not in (1) and (3).

Again, the statutory corporate income tax rate increases R&D expenditures only insignificantly in countries that do not offer a preferential tax rate on IP income, while it exerts a positive effect of some 2.5%, significant at the 5% significance level in countries that have an IP regime (net income approach) in place.

The heterogeneity we are interested in when estimating Eq. (7) concerns the new variables that indicate the application of the gross income approach. A significantly positive coefficient δ_2_ of Gross_approach*Stat_CIT would suggest that IP regimes with a gross approach help better to cushion the effects of a corporate tax increase than those with net approach. The fact that we observe only an insignificant positive coefficient is in line with the view that de facto, all IP boxes tend to be used as if they were following the gross income approach. This said, we should also keep in mind the limited observations that identify the size of the interaction effect and that limits statistical power.

If we concentrate on IP-regime countries with a gross approach in place, the coefficient representing the marginal effect of the statutory corporate income tax rate on the log of R&D expenditures is hardly changed (2.7%) compared to net approach countries but is not significant according to the test in line (iv) of Table 2.

Somewhat less expected, the coefficient on the interaction Gross_approach*τ_IP_, turns out positive and statistically significant at the 1% significance level in columns (2) and (4). It suggests that there is a difference on the effect of the preferential tax rate depending on whether a change in the tax rate happens in a gross or net IP regime approach. Lowering the preferential rate in a gross approach, which should be the more generous approach, seems to be less stimulating for R&D. Again, the fact that the estimation is based on tax rate change in only a few (here three) countries adds an important caveat.

## Conclusions

A growing literature indicates that high corporate taxes are detrimental to the number of patent applications by MNEs in these high-tax countries. Conversely, the question of whether high corporate taxes also reduce R&D expenditures and real research activity has received much less attention but is the focus of the present paper. We hope that this paper may trigger a larger discussion on taxes and the location of real R&D activities. While the location of patents may be informative on tax planning activities of MNEs, in the end, we expect that it is the location of real R&D activity that is decisive when it comes to international spillover effects in knowledge.

Using a model of R&D decisions by MNEs, we identified mechanisms that could induce more R&D expenditures when the tax rate increases. An intuition for this somewhat counter-intuitive tax effect is that R&D costs are tax deductible, and the value of this deduction tends to be the highest where the corporate tax is the highest. Given that R&D expenditures are tax deductible against the high corporate taxes, the possible positive R&D effect reflects a tax asymmetry: not all R&D returns are subject to the higher tax. First, since R&D creates a public good within the MNE, some of the R&D benefit is taxed at other countries’ tax rates that are not subject to the tax increase. Second, some of the R&D benefits are taxed at a lower IP regime tax rate. Therefore, a higher corporate tax, which increases value of the cost deductibility of R&D, may foster R&D. The intuition is related to the mechanics of the well-known debt tax shield, where a higher corporate tax rate makes the interest deduction more valuable. Our expectation is empirically supported by country-by-country R&D data of U.S.-owned subsidiaries for countries that do have an IP regime.

When it comes to the effect of IP regimes, we find a small overall impact on R&D expenditures, which is insignificant.

Several caveats and opportunities for future research remain. One issue is that our theoretical model is tailored to MNEs. It does not necessarily allow similar conclusions for national firms that conduct R&D. On the empirical side, one possible problem is that, as in the vast majority of papers evaluating the tax effects on patent behavior, we have taken changes in tax characteristics of countries as exogenous variations. While countries’ corporate tax rate decisions, much more than IP regimes, may be set with a focus on a broad set of goals, we cannot rule out that corporate taxes are set also with an eye to attracting R&D. At the same time, we did not find evidence that countries which introduced an IP regime also systematically changed their headline corporate tax.

Our empirical estimations are based on the R&D expenditures of U.S. wholly-owned subsidiaries, aggregated at the country-year level. Although the U.S. reports R&D for up to 75 different countries, confirming our results with confidential BEA firm-level data would be a worthwhile project, but would have to occur from within the BEA. Although subsidiary-level R&D expenditures are difficult to attain, using data from non-U.S. MNEs would also be a useful endeavor.

## Appendix A: Summary statistics and variables’ definitions

See (Tables 3, 4, 5).

**Table 3:** Summary statistics

Variable	Obs	Mean	Std. Dev	Min	Max
R&D expenditures ($mill)	621	697.15	1413.46	0	9133
LN (R&D expenditures)	588	4.36	2.66	0	9.12
Statutory Corporate income tax rate	621	24.09	7.13	0	40.69
Preferential tax rate on IP income	135	8.03	5.28	0	16.5
IP Regime	621	.22	.41	0	1
Gross approach	621	.04	.20	0	1
Expenditure-based Tax Incentives (EBTIs)	570	.63	.48	0	1
LN (GDP)	617	26.25	1.59	22.26	30.14
LN (GDP pC)	621	9.67	1.12	7.01	12.15
Unemployment	616	7.39	4.87	0	27.47
Corruption Perception Index	597	54.42	20.74	9	95
Property Rights Index	607	59.14	23.99	0	97.1
Trade	599	95.07	69.44	22.11	442.62

**Table 4:** Variables’ definition and data sources

R&D expenditures	Research and Development Expenditures of all U.S. Majority-owned Foreign Affiliates measured in millions of dollars, in country i in year t **Source:** Bureau of Economic Analysis, U.S. Department of Commerce
LN (R&D expenditures)	Natural logarithm of R&D expenditures **Source:** Bureau of Economic Analysis, U.S. Department of Commerce
Statutory CIT	Statutory Corporate Income Tax Rate **Source:** OECD Statistics database; KPMG International Corporate Tax Rates; EUROSTAT
Tax rate on IP income	Tax rate on IP income equals the Statutory corporate income tax rate if the host country of the U.S. MNE’s majority-wholly owned subsidiaries does not have an IP box regime (or similar) in a specific year, and thus taxes the income generated from the exploitation of IP at the normal statutory corporate income tax rate. Otherwise, if an IP regime is in place in country i, in year t, this variable is equal to the preferential (lower) tax rate applied to the IP income based on the IP box rules of that country in the specific year **Source:** For the Statutory CIT: OECD Statistics database; KPMG International Corporate Tax Rates; EUROSTAT; For the preferential tax rate on IP income: Initial orientation: OECD database on Intellectual Property Regimes; OECD (2015); OECD (2017); Atkinson and Andes (2011); Alstadsæter et al. (2015); Evers et al. (2015); Evers (2015); Sakar (2015); De Rassenfosse (2015); Guenther (2017); Schwab and Todtenhaupt (2017); Ernst and Young; European Commission, KPMG; PwC; Deloitte; National Website Sources
IP Regime (dummy)	IP Regime (dummy) takes on the value one if the host country of the U.S. MNE’s majority-wholly owned subsidiaries has an IP regime in place in the specific year and zero otherwise. Based on OECD (2015, 2017), classification as IP Regime refers to: (1) IP Regimes of OECD and G20 countries, (2) IP Regimes of new Inclusive Framework members, 3.) IP regimes of new inclusive framework members that are also reviewed as non IP regimes **Source:** OECD (2015, 2017); Atkinson and Andes (2011); Alstadsæter et al. (2015); Evers et al. (2015); Evers (2015); Sakar (2015); De Rassenfosse (2015); Guenther (2017); Schwab and Todtenhaupt (2017); Ernst and Young; European Commission; KPMG; PwC; Deloitte; National Website Sources
Gross approach (dummy)	Gross income approach takes on the value one if the country that has an IP regime in place applies an asymmetric treatment of IP income and IP expenses and as long as the taxpayer has sufficient ordinarily taxed non-IP income from which to deduct the IP expenses, this can produce substantial tax advantage. Thus, this variable takes on the value one if the current expenses are deductible from non-IP income, which is taxed at the regular corporate tax rate, and zero otherwise **Source:** Evers et al., (2014); Evers (2015)
Expenditure-based Tax Incentives (EBTI)	EBTI takes on the value one if a country offers at least one of the four R&D related tax incentives, namely tax credits, tax allowance, accelerated depreciation, and/or super deductions, and 0 otherwise **Source:** Ernst and Young Worldwide R&D incentives reference guides, PwC Worldwide Tax Summaries, OECD R&D Tax Incentives database, KPMG’s Europe, Middle East & Africa region (EMEA) research and development (R&D) incentives guide, as well as national websites
LN (GDP)	Natural logarithm of the GDP (in current $U.S.). GDP at purchaser's prices is the sum of gross value added by all resident producers in the economy plus any product taxes and minus any subsidies not included in the value of the products. It is calculated without making deductions for depreciation of fabricated assets or for depletion and degradation of natural resources **Source:** World Development Indicators from the World Bank database
LN (GDP Pc)	Natural logarithm of the GDP per capita (in $U.S.), expressed in GDP in PPP dollars per person. Data are derived by dividing GDP in PPP dollars by total population. These data form the basis for the country weights used to generate the World Economic Outlook country group composites for the domestic economy **Source:** World Development Indicators Database; International Monetary Fund; United Nations
Unemployment	Unemployment refers to the percentage share of the labor force that is without work but available for and seeking employment **Source**: World Bank Database
Corruption Perception Index (CPI)	Transparency International corruption index, which is constructed with higher values of the index indicating lower level of corruption **Source:** Transparency International
Property Rights Index	A subcomponent of the Index of Economic Freedom, the property rights index measures the degree to which a country’s laws protect private property rights, and the degree to which its government enforces these laws. The more certain the legal protection of property, the higher a country’s score; similarly, the greater the chances of government expropriation of property, the lower a country’s score **Source:** The Heritage Foundation, 2020 Index of Economic Freedom
Trade	Trade is the sum of exports and imports of goods and services measured as a share of gross domestic product **Source:** World Development Indicators form World Bank database

**Table 5:** List of IP regimes across countries

Country	IP regime	Existing before 2009/year of enactment	Name of regime	Preferential tax rate
Argentina	No			
Australia	No			
Austria	No			
Barbados	Yes	Existing before	International business companies	2.50%
Belarus	No			
Belgium	Yes	2007	Patents income deduction	6.80%
Bermuda	No			
Brazil	No			
Bulgaria	No			
Canada	No			
Chile	No			
China	Yes	Since 2008	Incentives for high and new technology enterprises (HNTEs)	15.00%
Colombia	Yes	2013	Software-Regime	0.00%
Costa Rica	No			
Croatia	No			
Czech Republic	No			
Denmark	No			
Dominican Rep	No			
Ecuador	No			
Egypt	No			
El Salvador	No			
Finland	No			
France	Yes	1971	Reduced rate for long term capital gains and profits from the licensing of IP rights	15.00%
Georgia	No			
Germany	No			
Ghana	No			
Greece	No			
Guatemala	No			
Honduras	No			
Hong Kong	No			
Hungary	Yes	2003, 2012	IP regime for royalties and capital gains	9.50%; 4.50%
India	Yes	2016	Patent-related incentive	10.00%
Indonesia	No			
Ireland	Yes	1973, 2008, 2016	Patent box, Knowledge development box	2.5%; 6.25%
Israel	Yes	Existing	IP regime (Preferred technology enterprise/ Special preferred technology enterprise status)	10.00%; 7.00%; 9.00%; 6.00%
Italy	Yes	2015	Taxation of income from intangible assets	13.95%
Jamaica	No			
Japan	No			
Korea, Republic of	Yes	2014	Special taxation for transfer, acquisition, etc., of technology	16.50%
Latvia	No			
Luxembourg	Yes	Since 2008	Partial exemption for income/gains derived from certain IP rights	5.84%
Macau	Yes	Existing before	Macau offshore institution	0.00%
Malaysia	No			
Mauritius	Yes	Existing before	Global business license 1(3%) and 2 (0%) regime	3.00%
Mexico	No			
Monaco	No			
Morocco	No			
Netherlands	Yes	2007, 2010	Patent box, Innovation box	5.00%
New Zealand	No			
Norway	No			
Oman	No			
Pakistan	No			
Panama	No			
Peru	No			
Philippines	No			
Poland	No			
Portugal	Yes	2014	Partial exemption for income from patents and other industrial property rights	15.00%; 10.50%
Qatar	No			
Romania	No			
Russia	No			
Saudi Arabia	No			
Singapore	Yes	2017	Intellectual property development incentive	10.00%
Slovakia	No			
Slovenia	No			
South Africa	No			
Spain	Yes	2008, 2013	Partial exemption for income from certain intangible assets (Federal regime)	15.00%; 12.00%
Sweden	No			
Switzerland	No			
Taiwan	No			
Thailand	Yes	Existing before	International headquarters regime/Regional operating headquarters regime	10.00%
Turkey	Yes	2015	Technology development zones regime	0.00%
United Kingdom	Yes	2013	Patent Box	10.00%
Uruguay	Yes	Existing before	Free Zones & Benefits under lit S art.52 for biotechnology and for software	0.00%
Venezuela	No			
Vietnam	No			

Classification as IP Regime refers to: 1.) IP Regimes of OECD and G20 countries, 2.) IP Regimes of new Inclusive Framework members, 3.) IP regimes of new inclusive framework members that are also reviewed as non-IP regimes. Some preferential regimes provide benefits to both income from IP and other non-IP, geographically mobile activities. These “dual category” regimes are reviewed as both an IP regime and a non-IP regime and therefore have to comply with both substantial activities requirements and two separate conclusions are applicable to the regime

Main sources: OECD (2015, 2017); OECD database on Intellectual Property Regimes

Other sources: Refer to Table A2

## Appendix B: Robustness tests

(See Tables 6, 7, 8, 9, 10).

**Table 6:** Additional control variables

Dependent variable: R&D Expenditures of U.S. majority-owned subsidiaries								
Model	OLS	OLS	OLS	OLS	NBM	NBM	NBM	NBM
	LN (R&D Expn.)	LN (R&D Expn.)	LN (R&D Expn.)	LN (R&D Expn.)	R&D Expn	R&D Expn	R&D Expn	R&D Expn
Regressors	(1)	(2)	(3)	(4)	(5)	(6)	(7)	(8)
IP Regime *(dummy)*	− 0.270	− 0.273	− 0.184	− 0.188	− 0.120	− 0.130	− 0.0636	− 0.0735
	(0.220)	(0.236)	(0.229)	(0.241)	(0.192)	(0.193)	(0.197)	(0.198)
Tax rate on IP income	− 0.0246**	− 0.0246**	− 0.0310***	− 0.0310***	− 0.0241**	− 0.0240**	− 0.0299**	− 0.0299**
	(0.0106)	(0.0105)	(0.0110)	(0.0110)	(0.0115)	(0.0115)	(0.0118)	(0.0118)
Statutory CIT	0.0367***	0.0366**	0.0425***	0.0423***	0.0421***	0.0416***	0.0473***	0.0467***
	(0.0137)	(0.0142)	(0.0147)	(0.0153)	(0.0147)	(0.0147)	(0.0156)	(0.0156)
IP Regime *(dummy)* * Statutory CIT	− 0.00865	− 0.00854	− 0.0167	− 0.0165	− 0.0110	− 0.0105	− 0.0173	− 0.0169
	(0.0121)	(0.0125)	(0.0126)	(0.0128)	(0.0122)	(0.0121)	(0.0129)	(0.0128)
Expenditure-based Tax Incentives (EBTIs)	− 0.365	− 0.365	− 0.369	− 0.369	− 0.179	− 0.180	− 0.182	− 0.183
	(0.262)	(0.260)	(0.262)	(0.261)	(0.193)	(0.194)	(0.194)	(0.194)
LN (GDP)	0.656*	0.657*	0.646*	0.646*	0.606**	0.606**	0.600**	0.600**
	(0.342)	(0.346)	(0.352)	(0.356)	(0.294)	(0.296)	(0.300)	(0.301)
LN (GDP pc)	0.191	0.191	0.193	0.193	0.0304	0.0333	0.0356	0.0389
	(0.380)	(0.380)	(0.388)	(0.388)	(0.293)	(0.293)	(0.298)	(0.297)
Unemployment	− 0.0304*	− 0.0304*	− 0.0308*	− 0.0309*	− 0.0308*	− 0.0308**	− 0.0303*	− 0.0304**
	(0.0175)	(0.0173)	(0.0178)	(0.0175)	(0.0157)	(0.0156)	(0.0156)	(0.0155)
Corruption perception index (CPI)	0.00394	0.00396	0.00366	0.00368	0.00190	0.00200	0.00162	0.00172
	(0.00307)	(0.00325)	(0.00303)	(0.00321)	(0.00321)	(0.00327)	(0.00326)	(0.00331)
Trade	0.000513	0.000521	0.000465	0.000483	− 0.000265	− 0.000259	− 0.000288	− 0.000281
	(0.00326)	(0.00329)	(0.00327)	(0.00330)	(0.00265)	(0.00265)	(0.00265)	(0.00266)
Gross approach *(dummy)*			− 0.551	− 0.560			− 0.354	− 0.374
			(0.514)	(0.514)			(0.459)	(0.453)
Gross approach *(dummy)* * Statutory CIT			0.00102	0.00126			− 0.0112	− 0.0106
			(0.0216)	(0.0215)			(0.0179)	(0.0177)
Gross approach *(dummy)* * Tax Rate on IP income			0.0475***	0.0477***			0.0544***	0.0547***
			(0.0160)	(0.0163)			(0.0205)	(0.0205)
Property Rights Index		− 0.000175		− 0.000379		− 0.000952		− 0.00107
		(0.00570)		(0.00573)		(0.00360)		(0.00359)
*Country FE*	Yes	Yes	Yes	Yes	Yes	Yes	Yes	Yes
*Time FE*	Yes	Yes	Yes	Yes	Yes	Yes	Yes	Yes
Obs	522	522	522	522	537	537	537	537
*R*^2^ (within)/Pseudo *R*^2^	0.376	0.376	0.378	0.378	0.30	0.30	0.30	0.30
Nr. of countries	64	64	64	64	64	64	64	64
Log pseudolikelihood					− 2578.7454	− 2578.6907	− 2577.9041	− 2577.8357
Alpha for overdispersion (std.error)					.05513 (.01201)	.05514 (.01200)	.05488 (.01196)	.0548933 (.01196)

The estimates add two control variables to those used in Table 1: Trade and Property Rights Index. The dependent variable in columns (1), (2) (3), and (4) is the log of R&D expenditures, and the number of R&D expenditures in columns (5), (6), (7), (8). The levels of significance are reported as ****p* < 0.01, ***p* < 0.05, **p* < 0.1. *p* values are based on robust standard errors, clustered at the country level. The model is estimated via OLS in regression (1), (2), (3), and (4). In columns (1)–(4) and (3)–(4), all the main variables of interest preserve their sign and their significance levels of Table 1 (column 1 and 2) and Table 2 (column 1 and 2). The model is estimated via a negative binomial model in regressions (5), (6), (7), (8). All estimations include country-fixed effects and time-fixed effects and countries are observed during the period 2009–2017 (unbalanced sample). The unit of observation is country-year. The alpha parameter informs about the degree of dispersion, if alpha is significantly greater than zero the data are over dispersed and are better estimated using a negative binomial model than Poisson models, which are reported in B2 below

**Table 7:** Poisson FE model

Dep.variable: R&D expenditures of U.S. majority-owned subsidiaries; Poisson FE model		
Regressors	(1)	(2)
	R&D Expn	R&D Expn
IP Regime *(dummy)*	− 0.0755 (0.140)	− 0.275** (0.114)
Tax rate on IP income	− 0.00492 (0.0130)	− 0.00201 (0.0142)
Statutory CIT	0.0257 (0.0159)	0.00904 (0.0144)
IP Regime *(dummy)* * Statutory CIT	0.000104 (0.00651)	0.00727 (0.0111)
Expenditure-based Tax Incentives (EBTIs)	0.0606 (0.135)	0.0351 (0.147)
LN (GDP)		0.520 (0.512)
LN (GDP pC)		0.358 (0.497)
Unemployment		− 0.0261 (0.0200)
Corruption Perception Index (CPI)		− 0.0124** (0.00582)
(i) τ_IP_it_ + Stat_CIT_i_	0.0208	0.0070
(ii) (1 + ipr_it_)*Stat_CIT_it_	0.0226*	0.0163*
(iii): (ii) – (i)	0.0050	0.0092
Obs	570	546
Nr. of countries	68	65
Log pseudolikelihood	− 9064.02	− 7354.91

The left hand variable in column (1) and (2) is the level of R&D expenditures. Coefficients derive from a panel Poisson estimator. The levels of significance, based on robust standard errors, are reported as ****p* < 0.01, ***p* < 0.05, **p* < 0.1. All estimations include country-fixed effects and time-fixed effects. Countries are observed during the period 2009–2017 (unbalanced sample). The unit of observation is country-year. As discussed in Footnote 12, the results of the OLS and NB models are not robust to using a Poisson model

**Table 8:** Estimation of Eqs. (6) and (7) using an Inverse Hyperbolic Sine Transformation

Dependent Variable: R&D Expenditures of U.S. majority-owned subsidiaries				
Model	OLS			
Regressors	(1)	(2)	(3)	(4)
IP Regime *(dummy)*	0.0270 (0.418)	− 0.285 (0.442)	0.271 (0.403)	− 0.142 (0.474)
Tax rate on IP income	− 0.0538** (0.0214)	− 0.0484** (0.0222)	− 0.0683*** (0.0200)	− 0.0603** (0.0231)
Statutory CIT	0.0932*** (0.0292)	0.0888*** (0.0274)	0.106*** (0.0292)	0.0999*** (0.0293)
IP Regime *(dummy)* * Statutory CIT	− 0.0384 (0.0257)	− 0.0241 (0.0260)	− 0.0583** (0.0243)	− 0.0384 (0.0277)
Expenditure − based Tax Incentives (EBTI)	− 0.542 (0.448)	− 0.706 (0.464)	− 0.547 (0.446)	− 0.711 (0.465)
LN (GDP)		1.070 (0.733)		1.058 (0.749)
LN (GDP pC)		0.398 (0.806)		0.402 (0.818)
Unemployment		− 0.0676** (0.0309)		− 0.0678** (0.0313)
Corruption Perception Index (CPI)		0.00473 (0.00562)		0.00421 (0.00563)
Gross approach *(dummy)*			− 1.644 (1.409)	− 0.878 (1.027)
Gross approach *(dummy)* * Statutory CIT			0.0175 (0.0494)	− 0.00829 (0.0407)
Gross approach *(dummy)* * Tax Rate on IP income			0.106 (0.0644)	0.0959*** (0.0319)
*Country FE*	Yes	Yes	Yes	Yes
*Time FE*	Yes	Yes	Yes	Yes
Observations	570	546	570	546
*R*-squared	0.299	0.382	0.301	0.383
Nr. of countries	68	65	68	65

This table reports results of the estimation of Eqs. (6) and (7) using an Inverse Hyperbolic Sine (HIS) Transformation of the dependent variable. Unlike in Tables 1 and 2, the HIS transformation keeps observations which have a zero value of R&D expenditures of U.S. majority-owned subsidiaries. Countries are observed during 2009–2017 (unbalanced sample). The levels of significance are reported as ****p* < 0.01, ***p* < 0.05, **p* < 0.1. p-values are based on robust standard errors, clustered at the country level. In column (2), all the coefficients on the main variables of interest preserve their sign, as well as their significance level, while they result to be higher compared to those reported in column (2) of Table 1, where the logarithmic transformation is used. In addition, using the HIS, the marginal effect of the statutory corporate income tax rate on R&D expenditures in countries without an IP regime in place becomes marginally significant. It remains positive and statistically significant at the 1% significance level in countries with an IP regime in place, although economically higher than the one using the logarithmic transformation

**Table 9:** Estimation of Eq. (6) using full sample of countries

Dependent Variable: R&D Expenditures of U.S. majority-owned subsidiaries				
Model	OLS	OLS	NBM	NBM
	LN (R&D Expn.)	LN (R&D Expn.)	R&D Expn	R&D Expn
Regressors	(1)	(2)	(3)	(4)
IP Regime *(dummy)* (ipr_it_)	− 0.156 (0.210)	− 0.318 (0.203)	− 0.0211 (0.195)	− 0.221 (0.187)
Tax rate on IP income (τ_IP_it_)	− 0.0294*** (0.0100)	− 0.0279*** (0.00983)	− 0.0276** (0.0111)	− 0.0265** (0.0103)
Statutory CIT (Stat_CIT_it_)	0.0391*** (0.0142)	0.0364*** (0.0137)	0.0417** (0.0165)	0.0340** (0.0159)
IP Regime *(dummy)* * Statutory CIT (ipr_it_ * Stat_CIT_it_)	− 0.0151 (0.0123)	− 0.00858 (0.0110)	− 0.0167 (0.0124)	− 0.00913 (0.0107)
LN (GDP)		0.703** (0.301)		0.611** (0.281)
LN (GDP pC)		0.0100 (0.348)		0.0382 (0.292)
Unemployment		− 0.0266* (0.0151)		− 0.0234 (0.0144)
Corruption Perception Index (CPI)		0.00615** (0.00308)		
		0.00346 (0.00336)		
(i) τ_IP_it_ + Stat_CIT_i_	0.0097 (0.293)	0.0085 (0.349)	0.0141 (0.241)	0.0075 (0.546)
(ii) (1 + ipr_it_)*Stat_CIT_it_	0.0241*** (0.005)	0.0278*** (0.004)	0.0249*** (0.006)	0.0249** (0.018)
(iii): (ii) – (i)	0.0141** (0.038)	0.0193** (0.011)	0.0109* (0.091)	0.0174** (0.013)
Obs	588	572	621	597
Nr. Of countries	75	72	75	72
R^2^ (within)/ Pseudo R^2^	0.30	0.36	0.30	0.31
Log pseudolikelihood			− 2846.15	− 2765.56
Alpha for overdispersion (std. error)			(0.0725) (0.0142)	(0.0615) (0.013)

The table omits the variable expenditure-based tax incentives (EBTIs) to increase sample size. The dependent variable in columns (1) and (2) is the log of R&D expenditures; in the negative binomial models of columns (3) and (4) R&D expenditures (in $mill) is used. Levels of significance: ****p* < 0.01, ***p* < 0.05, **p* < 0.1. *p *values are based on robust standard errors, clustered at the country level. Countries are observed during 2009–2017 (unbalanced sample). All estimations include country-fixed effects and time-fixed effects. The unit of observation is country-year. The alpha parameter informs about the degree of dispersion, if alpha is significantly greater than zero the data are over dispersed and are better estimated using a negative binomial model than a Poisson model

**Table 10:** Estimation of Eq. (7) using full sample of countries

Dependent Variable: R&D Expenditures of U.S. majority-owned subsidiaries				
Model	OLS	OLS	NBM	NBM
	LN (R&D Expn.)	LN (R&D Expn.)	R&D Expn	R&D Expn
Regressors	(1)	(2)	(3)	(4)
IP Regime *(dummy)*	− 0.00729 (0.169)	− 0.231 (0.201)	0.0945 (0.160)	− 0.148 (0.176)
Tax rate on IP income	− 0.0373*** (0.00905)	− 0.0343*** (0.0100)	− 0.0357*** (0.00981)	− 0.0328*** (0.0103)
Statutory CIT	0.0459*** (0.0140)	0.0424*** (0.0143)	0.0481*** (0.0161)	0.0395** (0.0163)
IP Regime *(dummy)* * Statutory CIT	− 0.0265*** (0.00998)	− 0.0167 (0.0109)	− 0.0271*** (0.0103)	− 0.0165 (0.0103)
Gross approach	− 0.996 (0.723)	− 0.537 (0.520)	− 0.771 (0.697)	− 0.468 (0.477)
Gross approach * Statutory CIT	0.0183 (0.0260)	0.00118 (0.0215)	0.00804 (0.0282)	− 0.00469 (0.0202)
Gross approach * Tax rate on IP income	0.0490 (0.0345)	0.0464*** (0.0163)	0.0526 (0.0456)	0.0513** (0.0234)
LN (GDP)		0.695** (0.308)		0.602** (0.288)
LN (GDP pC)		0.0112 (0.354)		0.0431 (0.298)
Unemployment		− 0.0269* (0.0153)		− 0.0232 (0.0142)
Corruption Perception Index (CPI)		0.00589* (0.00308)		0.00315 (0.00345)
(i) τ_IP_it_ + Stat_CIT_i_	0.0085	0.0080	0.012	0.0067
(ii) (1 + ipr_it_)*Stat_CIT_it_	0.019**	0.0256**	0.0209**	0.023**
(iii): (ii) – (i)	0.0107*	0.0176**	0.0086	0.016**
(iv) (1 + ipr_it +_ Gross approach)*Stat_CIT_it_	0.037	0.026	0.029	0.018
(v): (iv)–(ii)	0.018	0.012	0.008	− 0.0046
(vi) τ_IP_it_ (1 + Gross approach)	0.011	0.012	0.016	0.018
Obs	588	572	621	597
Nr. of countries	75	72	75	72
*R*^2^ (within)/ Pseudo *R*^2^	0.30	0.36	0.30	0.31
Log pseudolikelihood			− 2844.55	− 2764.64
Alpha for overdispersion (std. error)			(0.0720) (0.0140)	(0.0612) (0.0129)

The table omits the variable expenditure-based tax incentives (EBTIs) to increase sample size. The dependent variable in columns (1) and (2) is the log of R&D expenditures, in columns (3) and (4) R&D expenditures (in $mill). Levels of significance: ****p* < 0.01, ***p* < 0.05, **p* < 0.1. *p* values in parentheses are based on robust standard errors clustered at the country level. The model is estimated via OLS in regressions (1) and (2), and via a negative binomial model in (3) and (4). Countries are observed during the period 2009–2017 (unbalanced sample). All estimations include country-fixed effects and time-fixed effects. The unit of observation is country-year. The alpha parameter informs about the degree of dispersion, if alpha is significantly greater than zero the data are over dispersed and are better estimated using a negative binomial model than a Poisson model

## Footnotes

1 Recent evidence that corporate R&D leads to productivity gains in other parts of a multinational is provided by Bilir and Morales (2020). The idea that knowledge is shared across locations within the firm dates back at least to Arrow (1975) and is consistent with recent studies emphasizing the transfer of intangible inputs within firm boundaries as a key motive for plant integration (Atalay et al., 2014).

2 Other objectives that may motivate governments’ decisions to introduce IP regimes might be: to foster domestic innovation and the creation of high-value jobs; to incentivize firms to increase investment in innovative activities; to attract or retain mobile investments that may be associated with high-skilled jobs and knowledge creation (Bradley et al., 2015; Evers et al., 2015).

3 So far, theoretical contributions to the literature on IP regimes are rare. An exception is the study by Haufler and Schindler (2023) who look at IP regimes and R&D subsidies in a tax competition framework. They argue that IP regimes with their reduced rates on intellectual property are part of a non-cooperative equilibrium, but not part of a cooperative solution.

4 According to the BEA data description, R&D expenditures refer to expenditures for the planned, systematic pursuit of new knowledge or understanding toward general application (basic research); the acquisition of knowledge or understanding to meet a specific, recognized need (applied research); and the application of knowledge or understanding toward the production or improvement of a product, service, process, or method (development). It excludes quality control, routine product testing, market research, sales promotion, sales service, and other nontechnical activities; routine technical services; geological and geophysical exploration activities, and advertising programs to promote or demonstrate new products or processes. Also excluded are capital expenditures, expenditures for tests and evaluations once a prototype becomes a production model, patent expenses, and income taxes and interest.

5 The R&D expenditures reported in our data are attributed to the affiliate that conducts the real research activity. Therefore, any income from the patent(s) associated with the R&D expenditures reported in the respective country and year would satisfy the nexus requirement, if in place in that country. Consequently, we do not control for the nexus requirement.

6 Among the 163 countries listed in the database, R&D expenditures of U.S. MNEs are greater than 0, for at least one year between 2009 and 2017 in only 75 countries. For the rest of countries, data on R&D expenditures is missing for all the years or reported as 0 only for one or two years.

7 See Table 4 in Appendix A, for the definition of all variables used and the list of the data sources for each of them.

8 As robustness checks, in Tables 9 and 10, we exclude the expenditure-based tax incentives (EBTIs) dummy variable in order to provide the results for the full set of countries.

9 In 2011, the Canton of Nidwalden in Switzerland introduced a License Box Regime, which provided exemption at the cantonal level, but not at the federal level, equal to 80% of eligible income. Given that the tax advantage was not given at the federal level, in the regressions below, Switzerland is considered as a country without IP regime (Guenther, 2017, p.9). Switzerland recently introduced a patent box regime going into effect in 2020, which covers all of Switzerland. The regime provides a maximum tax base reduction of 90% on income from patents and similar rights developed in Switzerland. Cantons can opt for a lower reduction.

10 A robustness check in Appendix B estimates Eq. (6) and (7) by using an Inverse Hyperbolic Sine transformation of our dependent variable, which helps us to keep observations with zero R&D expenditures, which in the logarithmic form, turn out into missing values. Table 8 in Appendix B reports very similar results to Tables 1 and 2, although some coefficients of interest are slightly higher.

11 A Hausman test suggested that the fixed-effects model is more appropriate than a random-effects model.

12 Although this suggestion may have an incidental parameter problem, Allison and Waterman (2002) and Greene (2004) suggest that the resultant incidental parameters bias is not disturbing due to moderately small-time dimension (Cameron and Trivedi, 2013, p. 357). Note that our negative binomial model uses country dummies as suggested by Allison and Waterman (2002) that, unlike the conditional negative binomial model, accounts for all invariant country characteristics. An alternative to the NB model would be to use a Poisson model. However, our dependent variable has a variance of 1997869 and mean of 697, implying a ratio of 2867. The Likelihood ratio test is highly significant at the one percent level. In such a situation, we think that a Poisson model is not appropriate. The results of a Poisson model are not significant as shown in Appendix B, Table 7 and the estimates not only differ with respect to the negative binomial model but also with regard to the OLS FE model.

13 Presence of heteroscedasticity is suggested by scatterplots of fitted, predicted and residual values, Breusch–Pagan test, White test, and a modified Wald statistic for group-wise heteroscedasticity in the residuals of a fixed-effect regression model.

14 Note that changing the IP rate and holding the CIT rate constant is only possible for ipr_it_ = 1.

15 Spain moved from a ‘gross-income’ to a ‘net-income approach’ as part of the comprehensive reform of the IP Box implemented in September 2013 (Law 14/2013 of 27 September 2013), (Evers, 2015, p.71).
